# On the Post Hoc Explainability of Optimized Self-Organizing Reservoir Network for Action Recognition

**DOI:** 10.3390/s22051905

**Published:** 2022-03-01

**Authors:** Gin Chong Lee, Chu Kiong Loo

**Affiliations:** 1 Faculty of Engineering and Technology, Multimedia University, Jalan Ayer Keroh Lama, Melaka 75450, Malaysia; gclee@mmu.edu.my; 2 Department of Artificial Intelligence, Faculty of Computer Science and Information Technology, Universiti Malaya, Kuala Lumpur 50603, Malaysia

**Keywords:** echo state networks, action recognition, self-organizing networks, deep neural networks

## Abstract

This work proposes a novel unsupervised self-organizing network, called the Self-Organizing Convolutional Echo State Network (SO-ConvESN), for learning node centroids and interconnectivity maps compatible with the deterministic initialization of Echo State Network (ESN) input and reservoir weights, in the context of human action recognition (HAR). To ensure stability and echo state property in the reservoir, Recurrent Plots (RPs) and Recurrence Quantification Analysis (RQA) techniques are exploited for explainability and characterization of the reservoir dynamics and hence tuning ESN hyperparameters. The optimized self-organizing reservoirs are cascaded with a Convolutional Neural Network (CNN) to ensure that the activation of internal echo state representations (ESRs) echoes similar topological qualities and temporal features of the input time-series, and the CNN efficiently learns the dynamics and multiscale temporal features from the ESRs for action recognition. The hyperparameter optimization (HPO) algorithms are additionally adopted to optimize the CNN stage in SO-ConvESN. Experimental results on the HAR problem using several publicly available 3D-skeleton-based action datasets demonstrate the showcasing of the RPs and RQA technique in examining the explainability of reservoir dynamics for designing stable self-organizing reservoirs and the usefulness of implementing HPOs in SO-ConvESN for the HAR task. The proposed SO-ConvESN exhibits competitive recognition accuracy.

## 1. Introduction

Human action recognition (HAR) has been an active research field to interpret human intentions. HAR studies aim to develop real-world and reliable applications to perceive, study and identify human actions in videos [1]. The advancement in depth data acquisition hardware technologies such as the ASUS Xtion Pro or the Microsoft Kinect has led to the rise of noticeable HAR research outcomes in both commercial products and studies. Some prominent HAR applications include visual surveillance [2,3], human computer interaction [4,5], physical rehabilitation [6], and autonomous driving vehicles [7].

The simplicity and accuracy of extracting three-dimensional (3D)-skeleton-joints from depth images [8] have driven our work to focus on 3D-skeleton-joints-based HAR. The research works on 3D-skeleton-joints-based HAR still remains demanding. Current studies consider skeleton-joints human actions as multivariate time-series of five body parts (left arm, right arm, left leg, right leg and a central trunk) and attempt to identify and model the dynamical temporal features in 3D space. Echo State Networks (ESNs) [9] are such a popular Reservoir Computing (RC) method which is suitable for learning the temporal context. Its simplicity by randomly initializing and fixing the ESN’s input and reservoir weights during training, as compared with backpropagation through time of Recurrent Neural Networks (RNNs), diminishes the computational complexity. Furthermore, short-term memory property which ensures history information of the reservoir is not broadcasted to other neurons too rapidly, makes ESN suitable to capture the dynamical temporal features in HAR. Recently, Ma et al. [10] proposed an integrated architecture for HAR tasks, the Convolutional Echo State Network (ConvESN), by combining the RC with convolutional deep learning. Owing to ESN multiscale memory, echo state representations (ESRs) of input action series produced by ConvESN contains the history information which make it suitable to characterize and capture temporal dynamics in 3D skeleton series. Additionally, in ConvESN, the Convolutional Neural Network (CNN) [11] substitutes the linear regression in ESN output layer to understand the complex action echo states. In spite of ESN-based approaches achieved encouraging recognition performance, in this work we have identified three research avenues for our investigation: (1) Despite the random initialization of the ESN’s input and reservoir weights may reduce computational cost, on the other hand, this may rise instability and variance in generalization and hence diminish reproducibility [12]. In the context of HAR, a stable and reproducible multiscale feature extraction mechanism is needed to guarantee the performance of action recognition, in particular, when 3D skeleton joints are considered as multivariate time-series. Randomly fixed neuron weights may diversify the recognition performance of ESN-based approaches even in performing the same task with the identical set of hyperparameter configurations [13]. It hardly reproduces the same performance due to the randomized input and reservoir weights in different repeated runs. (2) Building an ESN model requires a set of hyperparameters to be configured. Hyperparameters are external to the model and are commonly tuned based on rule of thumb or empirically fixed via trial-and-error by researchers’ past experiences. Moreover, ESN remains as a black box algorithm. Particularly, it lacks of explainability consideration to understand the input-dependent reservoir dynamics for HAR. Using the explanatory information about the knowledge learned by ESN in tuning the hyperparameters seems to be promising. (3) Following the body of work of ConvESN, it has incorporated modelling dynamics and multiscale temporal feature in a unified framework. However, the model may be very sensitive to the selection of hyperparameters in CNN. Notably, ConvESN implemented Adam [14] optimizer in which the learning rate monotonically decreases based on the training iteration index. Careful selection of initial learning rate is required to alleviate training traps in local minima. Moreover, finding the optimal set of CNN hyperparameters can become computational costly [15], particularly, ConvESN implemented manual hyperparameter tuning for CNN stage.

To address these problems, in this work we propose a novel reservoir design approach known as the Self-Organizing Reservoir Network with Explainability (SORN-E) which is characterised by (i) the integration of Adaptive Resonance Theory (ART) [16] architecture and topology construction based on Instantaneous Topological Mapping (ITM) [17] for the self-organization of the input weights and reservoir weights, and ii) hyperparameter tuning based on the explainability of self-organizing reservoir through Recurrent Plots (RPs) and Recurrence Quantification Analysis (RQA) technique [18]. Input-driven and self-organization have been proven to be crucial for the cortex to adapt the neurons in accordance with input topology or distribution [19]. This previous effort motivates our work to take unsupervised self-organizing learning into account as a potential biologically plausible approach for self-organizing reservoir design. Combining the advantages of ART and ITM, SORN-E has similar network architecture to handle plasticity and stability dilemma and the number of network nodes is not required to be defined prior the learning. The unsupervised learning process of SORN-E is composed of best-matching node selection, vigilance test, and node learning. SORN-E encodes human actions as multivariate time-series signals. It performs unsupervised learning from training dataset to generates a self-organizing clustered topology of nodes which maintains the topological properties of the input at a greatly reduced dimensionality. The generated maps are represented by clustered node centroids and interconnectivity maps. Input samples with sufficiently high similarity are often characterized by a single node or a cluster of nodes.

Furthermore, to guarantee stability and echo state property (ESP) [9], we exploit a state-of-the-art qualitative stability criterion for reservoirs of ESN called maximum diagonal line length, $L_{MAX}$ [18]. This RQA qualitative metric is used to quantify the reservoir stability. By measuring this metric at different configurations of input scaling ($I_{S}$) and spectral radius ($S_{R}$) of the reservoir, the optimal values for stability can be identified. With the aim to provide explanatory information of the dynamics and improve insights of SORN-E, RPs and RQA are used as descriptive approach to examine the dynamics of self-organizing reservoir and to gain a clear idea of the echo state representations (ESRs).

Referring to the body of work of ConvESN, the proposed SORN-E is cascaded with a simple CNN. The feature maps generated by SORN-E are applied to initialize the input weights and recurrent hidden weights in the ESN to yield optimized self-organizing reservoirs. CNN then learns the multiscale temporal features from ESRs for action recognition. This resultant novel implementation is named Self-Organizing Convolutional Echo State Network (SO-ConvESN). With respect to the learning of the CNN stage of the proposed approach, sequential, parallel, sequential-parallel hyperparameter optimization (HPO) algorithms are investigated with the intention of obtaining optimal HAR performance. SO-ConvESN has also been deployed to HAR tasks to demonstrate the feasibility and applicability.

In a nutshell, our main contributions can be summarized as follows:

- We propose an unsupervised self-organizing network for learning node centroids and interconnectivity maps which are compatible for the deterministic initialization of ESN reservoir weights. To ensure stability and ESP in a self-organizing reservoir, we further exploit the RQA technique for explainability and characterization of the dynamics of self-organizing reservoir and hence tuning two critical ESN hyperparameters: input scaling ($I_{S}$) and spectral radius ($S_{R}$).
- Cascading the stable and optimized self-organizing reservoirs with a simple CNN. Self-organizing reservoir ensures that the activation of ESN internal ESRs echoes similar topological qualities and temporal features of the input time-series and CNN efficiently learns the dynamics and multi-scale temporal features from the ESRs for action recognition.
- Adopting three different categories of HPO algorithms, namely Sequential method: Bayesian Optimization (BO) [20], Parallel method: Asynchronous Successive Halving Algorithm (ASHA) [21], and Parallel-Sequential method: Population-based Training (PBT) [22] to search for optimal hypermeters of CNN stage in SO-ConvESN for HAR tasks.
- Conducting experiments by using several publicly available 3D-skeleton-based action recognition datasets to examine the explainability of self-organizing reservoirs dynamics, investigate the recognition accuracy of SO-ConvESN and the feasibility of implementing HPOs in SO-ConvESN for the HAR task.

The rest of the paper is organized as follows: Reviews of the related works on ESN-based approaches for 3D-skeleton-based HAR and self-organizing approaches for clustering are first presented followed by the concise descriptions of explainability methods for ESN and HPO for CNN. Next, we describe the development framework of SO-ConvESN and the details of each stage: SORN-E, explainability methods, and HPO. The simulation experiments and results are then presented based on several publicly available benchmarking datasets. Last of all, concluding remarks are presented.

## 2. Related Works

Before proceeding further, this section first reviews the existing methods for HAR tasks, particularly the ESN-based approaches from which we identify suitable for 3D-skeleton-based HAR, and highlights the method’s limitations. We note that self-organization potentially fits reservoir design; we next highlight the overview of unsupervised self-organizing learning approaches. Finally, we provide a brief discussion of techniques for explainability of reservoir design and hyperparameter optimization of CNN.

### 2.1. ESN-Based Approaches for HAR Tasks

Current studies on 3D-skeleton-joints-based HAR making use of machine learning techniques, such as Support Vector Machines [23,24,25], Multilayer Perceptrons [25], Dynamic Time Warping [26,27,28], Hidden Markov Models [27], and Decision Trees [24,25] irregularly neglect the temporal features of human actions in 3D space. Temporal features could be containing helpful information over the intervals between activities. This problem drives the study of the HAR approaches to adequately modeling the temporal features in human actions data. Adopting memory mechanisms like the implementation in RNNs [29] is practical in handling temporal data for HAR. However, due to the gradient-based training of RNN architectures with numerous layers, it is well known to be experiencing the consequences of exploding or vanishing gradients. Long Short-Term Memory (LSTM) [30] networks address this error-prone gradient-based training by coupling gating mechanisms into RNN architecture and successfully enhancing the learning process of the temporal features for 3D-skeleton-joints-based HAR.

On the other hand, RC emerges as an alternative approach for conventional RNN training. ESNs [9] are a widespread special RC implementation for training RNN. The main characteristics that make ESNs distinguished are the short-term memory property contributed by the sparsely connected reservoir and reduced computational complexity compared to backpropagation through time yielded by the random initialization of the input and reservoir weights. These make ESNs a suitable method that fits for handling temporal context. Nevertheless, to the best knowledge of the literature review, the implementation of the ESN-based approach in HAR using 3D-skeleton joints has not been fully explored. Significatory strategies include bidirectional Leaky Integrator ESNs (LI-ESNs) [31], canonical ESN by integrating 3D body joints and objects category [13], and ConvESN [10]. Bidirectional LI-ESNs [31] perform direct processing on temporal 3D body joints without extra feature extraction procedures during the encoding process. Each input sequence must be completely available and go through the bidirectional reservoir to produce better state representation. Like canonical training-free ESN, LI-ESNs apply random initialization of input weights but describe a permutation matrix for the recurrent weights. On the other hand, Mici et al. [13] integrated additional contextual ideas with 3D body joints to enhance the performance of the HAR. This approach considers the participation between the performed action and the manipulated object to tackle ambiguities during the activity. Hence, the learning of ESN needs to combine the 3D body joints and the object labels. Object labels may impact the internal reservoir representation. Mici et al. [13] demonstrated the proposed approach to handle a multi-label classification task considering the amount and variety of manipulated objects that change in different actions. Mici et al. [13] also takes the advantage of randomly initialized and fixed input weight and recurrent weight in the reservoir.

The approaches mentioned above focused on the manipulation of mapping input data into the reservoir. However, linear regression, as in canonical ESN, may limit the decoding capability in the output layer [32]. Moreover, extracting dynamical, locally related features in 3D skeleton sequences is crucial to improve HAR performance. Existing feature extraction approaches in HAR by a heuristic handcrafted mechanism could be insufficient [33,34]. It greatly depends on researchers’ experience and domain knowledge. Ma et al. [10] introduced ConvESN as another ESN-based approach for 3D-skeleton-joints-based HAR to address these problems. ConvESN significantly impacted our works positively due to its superior structures in extracting multiscale temporal features from human actions data. In recent years, HAR research has applied deep learning, particularly CNN, for superior and automated high-level feature extraction [35]. The ConvESN is such an approach that bridged CNN and RC areas for the HAR task. It substitutes the ESN readout layer with a CNN that consists of a convolution layer followed by a max-pooling layer. By treating 3D-skeleton-joints input as multivariate time-series and projecting the series onto reservoir to generate ESRs, CNN effectively decodes the ESRs at all time steps and extracts dynamics and multiscale features. Ma et al. [10] demonstrated the effectiveness of ConvESN for HAR. In ConvESN, we can view the significance of CNN as complementary to the ESN stage in HAR. CNN decodes the multiscale features, which may be lacking by the ESN stage.

The ultimate goal of implementing ESN in the HAR approaches, as mentioned earlier, is to reduce the computational cost. However, the random initialization of the ESN’s reservoir may generate uncertainty and diversity in generalization. This variance may weaken reproducibility [12]. In a nutshell, repeating the same task with the identical set of hyperparameter configurations hardly reproduces the same recognition performance due to the randomly initialized and fixed neuron weights [13]. Palangi et al. [36] have shown that adapting one or both ESN’s input and recurrent weights to the input data offers more reliable classification performance. In biological analogy, self-organization and input-driven learning mechanism are also critical for the cortex to adapt the weights of the neurons to the input properties [19]. These notions show that adapting weights to the input data seems to be desirable. Besides, most of the existing ESN-based HAR methods also lack consideration of explainability in expressing the input-dependent reservoir dynamics. ESN often stays as a black-box algorithm. Expressing and understanding the explanatory information about the knowledge learned by ESN, especially reservoir echo state, could be helpful to set ESN’s hyperparameters configuration [37]. Focusing on the ConvESN, careful selection of hyperparameters in CNN is essential. During CNN weights learning, poor selection in the initial learning rate of the optimizer may leave the learning process stuck at local minima or saddle points. Currently, ConvESN implements manual hyperparameter tuning for the CNN stage. Finding the optimal set of CNN hyperparameters can be computationally costly [15].

With the issues mentioned above, we hypothesize that implementing a deterministic and self-organizing reservoir in ESN may ensure the activation of internal ESRs echoes similar topological qualities and temporal features of the input time-series. The explainability and characterization of the dynamics of the reservoir could also be helpful in tuning the ESN hyperparameters. Lastly, applying the HPO algorithms during CNN training could improve the HAR performance to a certain extent. We next extend the discussion to include the overview of unsupervised self-organizing learning approaches and a brief introduction of techniques for explainability of reservoir design and hyperparameter optimization of CNN.

### 2.2. Unsupervised Self-Organizing Learning Approaches

More recently, Kohonen[38] introduced an approach to initialize the canonical ESN’s reservoir based on unsupervised learning strategies. In essence, this approach applied Self-Organizing Map (SOM) [39] and Neural Gas Network (NG) [40,41] for the adaption of the reservoir input weights. The reservoir weight matrix was also learned from NG. However, the robustness of this approach was influenced by the dimensional of the input space. This limitation could be due to SOM and NG being characterized by a predefined and fixed-size topological map that cannot be changed over time. Growing Neural Gas (GNG) [42] has been introduced as another important algorithm of topological self-organizing clustering to address this limitation. GNG has extended the adaptive abilities to add new neurons, create and delete connections between neurons. SOM, NG, and GNG heavily assume that the input data are statistically independent. In particular, the GNG network grows at a fixed rate regardless of the variation of the input distribution. The limitation makes them challenging in manipulating trajectory data, such as 3D-skeleton joints. ITM [17] represents one approach to overcome this constraint. It handles input data with strong correlation by adapting a formation of a topological map. On the other hand, SOM, NG, and GNG algorithms also experience from plasticity-stability dilemma [43]. ART [16] is a well-known self-organizing approach that is capable to address this dilemma. The ART network has similar architecture as the learning process in the human cortex. Proposing a novel self-organizing reservoir design based on the integration of ART and ITM for the context of HAR seems to be motivating.

### 2.3. Explainability Methods for ESN

When developing an ESN for particular applications, respectively, for the HAR task, it is crucial knowing if the generated reservoir is optimal for the problem at hand. The area of explainability developed for ESN has been studied but at a knowingly smaller collection of published works. When developing an ESN for the particular applications, respectively, for the HAR task, it is crucial knowing if the generated reservoir is optimal for the problem at hand. Interpreting the reservoir’s explanatory information could improve and guide the development of ESN [37]. Bianchi et al. [18] address this issue by applying RPs [44,45,46] and heuristic complexity measures known as RQA to analyze the dynamics of the neuron activations in a reservoir. This technique is used as analysis tool to visualize and characterize the reservoir dynamics based on the echo state matrix. Alternatively, under the Explainable Artificial Intelligence (xAI) paradigm, Arrieta et al. [37] proposed a set of novel post hoc xAI techniques to discover the strengths and weaknesses for ESN-based models. It focuses on the explainability of Deep Echo State Networks (Deep ESNs) [47]. Comparing both mentioned techniques, RPs and RQA-based techniques generally seem to be well-suited for guiding self-organizing reservoir design. The reasons are twofold: RQA measure provides the stability criteria that are crucial to designing a stable reservoir. Moreover, RPs can be used to visualize the reservoir dynamics for better explainability. This work utilizes RPs and RQA-based techniques to extract the input-driven explanatory information captured over time for understanding self-organizing reservoirs and assess the reservoir stability properties for tuning the ESN hyperparameters. We include the comprehensive discussion of the RPs and RQA-based procedures to guide the design of the self-organizing reservoir in the later section. Applying explainability technique can provide transparency to understand and express the reservoir dynamics and use it in tuning the ESN’s hyperparameters configuration.

### 2.4. HPO Algorithms for CNN Training

As mentioned earlier, our work integrates SORN-E with CNN to yield SO-ConvESN to address the HAR task. Similar to most machine learning models, CNN possesses a set of external network configurations known as hyperparameters [48] that influence the learning process. Hyperparameters cannot be estimated or learned from the training data and must be carefully tuned to unlock the network performance fully. However, selecting the suitable configurations of the hyperparameters could be challenging. Some existing CNN models apply default implementation, which could not be optimal for the problem on hand. Besides, manually finding the optimal set of CNN hyperparameters, such as ConvESN, is common. The manual search process may require thorough hyperparameter tuning experiments that could be computationally costly [15]. In the worst case, the network training suffers from the sensitivity to the poor selection of some hyperparameters, such as the initial learning rate, which may influence the performance to be inadequate. Therefore, applying HPO may help reduce human efforts to search for the ideal CNN hyperparameters in an automated and structured manner.

Hyperparameters of CNN are often viewed as the variable of the network structure in which finding the optimal configurations leads to an optimization problem [15]. There is a wide variety of works on algorithms for HPO, which are often problem-dependent, and there is no single HPO method that suits all models [49,50,51,52]. We limit our focus on the well-established or/and state-of-the-art algorithms well-suited for CNN hyperparameter tuning. In general, two main categories of hyperparameter tuning algorithms exist: sequential methods and parallel methods [22]. These methods are distinguished by the use of computational resources and time elapsed to reach optimal configuration.

Sequential methods run several training processes consecutively. It progressively performs hyperparameter tuning. Each run uses information from the earlier training process to select new hyperparameters and retrain the model in the subsequent new runs. Typically, sequential methods employ minimum computational resources but require high hyperparameter tuning time. BO [20] is a sequential method that adaptively selects configurations by identifying proper search space to discover candidate hyperparameters to evaluate next. The inherently sequential optimization of BO makes this method commonly used for the small-scale regime. As an indicative algorithm in sequential methods, exploring and investigating the feasibility of BO in optimizing the hyperparameters of CNN in SO-ConvESN could be worth trying.

On the other side of the coin, parallel methods initialize network weights of multiple training processes and run with different hyperparameters in parallel. Parallel methods aim to obtain one single best-performing configuration from the multiple HPO processes. Generally, hyperparameter tuning time of parallel methods consume only one training process but employ more computational resources. Grid search and random search are representations of parallel HPO methods [49]. One of the state-of-the-art parallel methods is known as ASHA [21]. ASHA proposed a parallelization scheme by combining adaptive configuration selection as well as evaluation. It primarily addressed the issues of parallelizing Successive Halving Algorithm (SHA) in which the optimization time scales linearly with the number of workers [53]. ASHA asynchronously allocates more computational resources to the promising configuration and performs early stopping on underperformed training processes whenever possible to minimize stragglers. The parallelism of ASHA makes this method suitable for massive parallel optimization processes that require aggressive early stopping. Implementing ASHA could minimize the common overfitting problem in neural networks and hence suits for optimizing the hyperparameters of CNN in SO-ConvESN.

Recently, Population-Based Training (PBT) [22] has been introduced as a state-of-the-art HPO method that links and enhances parallel methods and sequential methods. PBT is similar to parallel methods [49]. It begins training by randomly selecting hyperparameters and initializing network weights. Additionally, it maintains a population of asynchronous training processes, periodically evaluated based on the chosen hyperparameters. In particular, it implements a novel explore-exploit paradigm to allocate the computational resources to the training process, which has the highest possibility to produce good performance meanwhile adaptively tune the hyperparameters during training. In PBT, an underperformed population member exploits other members of the population and replaces itself with a member with better performance. It also explores new hyperparameters to modify the hyperparameters of the better performing member. PBT is designed initially for neural networks. The jointly tuning of weight parameters and hyperparameters make PBT suited for the HPO of CNN.

Based on the potentials of each of the abovementioned techniques, it is interesting to implement these HPO algorithms and investigate the analysis results in optimizing the CNN to discover the most suitable method. Notably, in this work, applying these HPO algorithms during CNN training of SO-ConvESN, to a certain extent, could improve HAR performance.

## 3. Materials and Methods

This section first presents the overview of the development framework of SO-ConvESN for HAR. The paradigm of the proposed SORN-E approach and the approach of cascading SORN-E with a CNN to yield SO-ConvESN is then thoroughly introduced.

### 3.1. Development Framework of the Proposed SO-ConvESN

**Figure 1:**
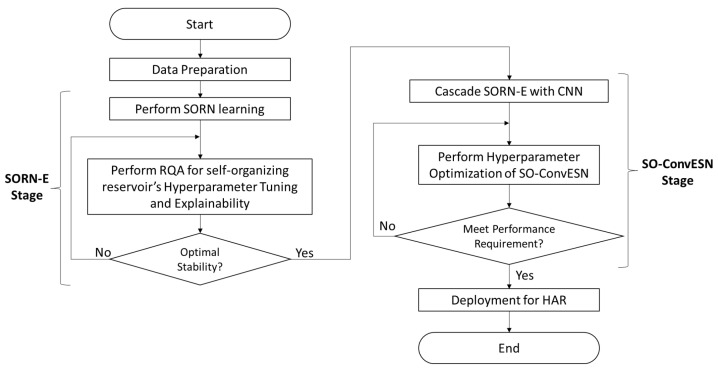
Development framework of the Proposed SO-ConvESN for HAR. It consists of two key components: the SORN-E stage and the SO-ConvESN stage.

This work aims to propose a novel ESN-based network known as SO-ConvESN, tackling HAR task based on 3D-skeleton single-view and single-person-based scenario. Two key components: SORN-E stage and SO-ConvESN stage, play crucial roles in the framework. Figure 1 depicts the overview of the workflow used to develop the proposed SO-ConvESN.

During data preparation, each skeleton series from training set is separated into five individual channels correspond to 3D-skeleton joint coordinate trajectories of five body parts; left arm (LA), right arm (RA), central trunk (CT), left leg (LL) and right leg (RL). Considering that the movement of head coordinates are synchronized with the movement of center of shoulders, this work discarded the head coordinates. Each of the body parts of the 3D skeleton joint was treated as multivariate time-series. They were used to train and construct the corresponding reservoirs. Self-organizing clustering was performed by SORN separately for each channel and the corresponding reservoir was created to obtain the action echo states. RQA is then implemented on the echo states to tune the hyperparameters of self-organizing reservoir for optimal stability. This process will be iterated until the satisfied stability is achieved.

Following the direction of ConvESN, the developed SORN-E is afterward cascaded with a simple CNN to formulate the SO-ConvESN. Optimized self-organizing reservoirs generates the ESRs from the corresponding channels. CNN then extracts the multiscale temporal features from these ESRs. Hyperparameter optimization algorithms are applied for searching the optimal hyperparameters of the CNN. Optimized SO-ConvESN is then deployed for HAR applications.

### 3.2. Self-Organizing Reservoir Network with Explainability (SORN-E)

**Figure 2:**
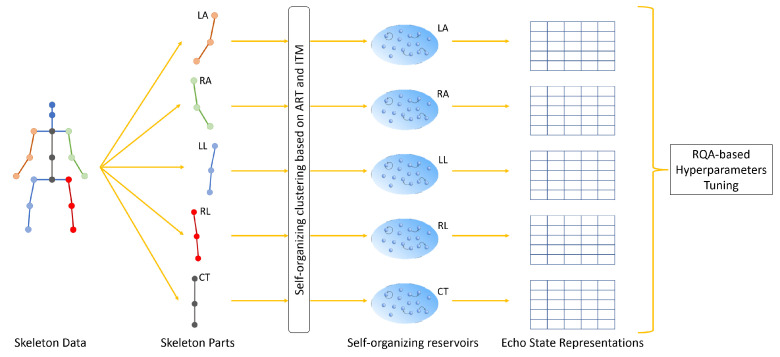
Five channels from human skeleton data: left arm (LA), right arm (RA), left leg (LL), right leg (RL), and central trunk (CT) are first extracted during data preparation. Then, SORN learning generates the respective self-organizing reservoirs independently. RQA is applied on the corresponding ESRs for hyperparameter tuning.

This section introduces the SORN-E for self-organizing reservoir design via the exploitation of explainability techniques. The proposed approach consists of two phases: Self-Organizing Reservoir Network (SORN) learning and RQA-based hyperparameter tuning. Both steps are applied separately to each of the five channels corresponding to the body parts over time. Figure 2 shows SORN generates five self-organizing reservoirs by clustering the five channels of skeleton data, followed by the implementing RQA on ESRs of each channel.

#### 3.2.1. First Phase: The Self-Organizing Reservoir Network (SORN) Learning

SORN has similar architecture as the ART network and an ITM-based topology construction process. SORN learning aims to learn a set of node centroids and interconnectivity maps from input skeleton data. During the topology construction process, SORN implements edge adaptation of ITM and the least-recently-used node pruning policy. Topological connections, the edges, link the nodes with similar neighboring information and form the cluster of nodes. In a nutshell, the SORN learning algorithm is composed of (i) best-matching node selection, (ii) node matching using vigilance test, (iii) node learning, and (iv) topology construction.

First of all, let us represent the 3D skeleton joint coordinates of a channel at a time instant, *t* as u(t). In order to accelerate the convergence of SORN clustering [54], noise is added to the input a priori according to Equation (1).

(1) $$z\left(t\right)=u\left(t\right)+\left(t^{-2}\eta \right)I$$

The degree of the noise is governed by η=[0,1]. *I* is an identity matrix and it is same size as u(t). The noise in z(t) would gradually reduce over time, *t*. The feasibility of designing self-organizing reservoirs using SORN is explored and analyzed in this work by going through:

- *Best-matching node selection.* The learning process starts with a new and empty SORN. Each noisy input z(t) at instant *t* is inserted into the SORN for self-organizing learning. For an empty SORN, z(t) is inserted as a new node as
  (2) K←K+1
  (3) $$w_{K}=z\left(t\right)$$
  where weight, $w_{K}$ is the weight of node *K*. Otherwise, the best matching node *b* and the second-best matching node *s* are selected based on the similarity comparison with z(t) as follows
  (4) $$k_{l}\left(z\left(t\right),w_{j}\right)={∥z\left(t\right)-w_{j}∥}^{2}$$
  (5) $$b=\underset{j\in J}{\mathrm{argmin}}\left[k_{l}\left(z\left(t\right),C\right)\right]$$
  (6) $$s=\underset{j\in J,j\neq b}{\mathrm{argmin}}\left[k_{l}\left(z\left(t\right),C\right)\right]$$
  $k_{l}$ is the Euclidean distance measured between the z(t) and a node *j* with weight, $w_{j}$, both *b* and *s* are the indices of the best matching node and the second-best matching node respectively, and *C* is the self-organizing clustered centroids with *J* nodes. SORN selects the second-best matching node for the later ITM-based topology construction process.
- *Node matching using vigilance test.* Next, a node matching between sample z(t) and node b will be succeeding the nomination of the best-matching node. The vigilance test evaluates if the sample z(t) stays inside the vigilance region of node *b* as follows
  (7) $$k_{l}\left(z\left(t\right),w_{b}\right)\leq V$$
  where *V* is the vigilance threshold. Using Hebbian rules, it decides whether to perform node learning or add a new node. If the sample z(t) and current node *b* do not satisfy the vigilance test, SORN will nominate the next candidate of the best matching node to fulfill the condition in Equation (7). If all the current *J* nodes fail the vigilance test, a sample z(t) is added as a new node into the SORN following Equations (2) and (3).
- *Node learning.* On the other hand, if the sample z(t) satisfies the vigilance test in Equation (7), then SORN performs node learning to update the weight of the best-matching node as follows
  (8) $$w_{b}=w_{b}+\epsilon_{b}\left(z\left(t\right)-w_{b}\right)$$
  where $\epsilon_{b}$ is the learning rate.
- *Topology construction.* After node learning, if the second-best matching node satisfies the matching condition in Equation (7), incrementing edges between the best-matching node *b* and second-best matching node *s* will be created if it does not exist priorly, as follows:
  (9) ΔE(b,s)=1.
  SORN constructs the interconnectivity matrix *E*, which defines the topology of nodes. Taking advantage of ITM, creating a topological map does not require the implementation of edge aging. After creating the connecting edge between node *b* and node *s*, vigilance region through node *b* and node *s* is compared with vigilance region through node *b* and every *n*-th member of *N* neighborhood nodes of best-matching node *b*, as follows:
  (10) $$k_{l}\left(w_{b},w_{s}\right)\leq k_{l}\left(w_{b},w_{n}\right).$$
  Suppose the vigilance region comparison in Equation (10) is satisfied; it implies that node *s* has a closer neighborhood to node *b*. Hence, edge deletion removes the connecting edge between node *n* and node *b*. For every λ learning cycle, node pruning based on least-recently-used policy prunes any node which does not connect to any topological edges. Although SORN does not require us to predefine the number of nodes, *N*, before the learning, we use it as the stopping criterion for generating a self-organizing reservoir with *N* neurons.

The SORN learning process stimulates the formation of a cluster of neighboring nodes that are more specialized and activated by a specific input skeleton data, meanwhile maintaining a certain degree of diversity of the dynamical behavior represented by a different cluster of more distant nodes. SORN defines the topological structure of the reservoir via the formation of interconnectivity maps *E*. Each neuron interacts with the neighbors according to interconnectivity maps *E*. It could be a recipe that is compatible with adapting ESN reservoir weights [38]. On the other hand, node centroids adapt the reservoir input weights from input skeleton data. Node centroids, up to a certain extent, capture the topological distribution of the input patterns. Similar input stimuli tend to activate the same cluster of neighboring nodes. Pre-establishing the ESN input weights with node centroids by SORN learning may form the action cluster in the ESN reservoir [38].

Upon SORN generates node centroids *C* and the interconnectivity maps *E*, we adopt these weights for the deterministic initialization of the reservoir parameters: input weights, $W^{in}$ and recurrent hidden weights, $W^{res}$. Input weights and recurrent hidden weights are crucial elements for the existence of echo states and the emergence of suitable dynamics. It is essential to accomplish a few adjustments in these ESN weights during the creation of the self-organizing reservoir. The rescaling procedure is similar to canonical ESN [9]. Firstly, clustered node centroid weights *C* are normalized and adjusted by the hyperparameter known as input scaling, $I_{S}$. We rescale each *j*-th element of node centroid weights to range between $-I_{S}$ to $I_{S}$ as in Equation (11) for j∈J.

(11) $$c_{j}=I_{S}.\left(2\left[\frac{c_{j}-min\left(C\right)}{max(C)-min(C)}\right]-1\right)$$

In the meantime, for the interconnectivity matrix *E*, we rescale it by the hyperparameter known as spectral radius, $S_{R}$ as in Equation (12).

(12) $$E_{rescaled}=S_{R}\frac{E}{\lambda_{max}\left(E\right)}$$

$\lambda_{max}\left(E\right)$ is the largest eigenvalue of interconnectivity matrix *E*. We adapt input weights and recurrent weights using Equations (13) and (14) to yield a self-organizing reservoir with *N* neurons.

(13) $$W^{in}=C$$

(14) $$W^{res}=E_{rescaled}$$

Rather than randomly fixed as in canonical ESN, SORN deterministically initializes the reservoir’s input weights and recurrent weights. This reservoir acting as a feature map, generates internal echo states that represent the similar topological conditions of the input action series and yield self-organization.

#### 3.2.2. Second Phase: The RQA-Based Hyperparameter Tuning

The self-organizing reservoirs generated by SORN dramatically depend on the configurations of input scaling $I_{S}$ and spectral radius $S_{R}$. Unfortunately, the exemplary configuration of these two crucial hyperparameters could often be relying on luck and require the experience of the researchers [12]. Mainly, literature works usually highlight that we must set $S_{R}$ close to but less than 1 to ensure the ESP [9]. It is necessary to have systematic guidance for hyperparameter tuning and a technique well-matched to express the explainability of the sound reasoning of already trained SORN. Given the above context, in this work, we demonstrate the applicability and showcasing of the RQA technique in designing stable self-organizing reservoirs and, by this means, properly facilitate the hyperparameters configuration process for the problem at hand. Combining SORN with the RQA technique to tune self-organizing reservoir hyperparameters and provide post hoc explainability led to the reservoir design approach we called SORN-E.

RQA technique requires building RPs and measurement criteria for tuning reservoir hyperparameters and supporting the explainability of the self-organizing reservoir. Therefore, after SORN learning and generating the self-organizing reservoirs, we construct RPs from ESRs and define the necessary measures.

Assuming u(t) represents a *D*-dimensional 3D skeleton joint coordinates of a single body part at a single time instance *t* and $x\left(0\right)\in R^{N}$ is an initial echo-state in the self-organizing reservoir. Similar to canonical ESN, projecting the time-series input at time instant t∈[0,T−1] into the self-organizing reservoir generates an echo state according to the updated equation as follows:

(15) $$x\left(t+1\right)=f(W^{res}x\left(t\right)+W^{in}u\left(t+1\right)).$$

For the *T*-length time-series input, ESRs are represented by an *N*-by-*T* dimensional matrix as follows:

(16) $$X={\left(x\left(0\right),\ldots ,x\left(T-1\right)\right)}^{T},$$

where an ESR state x(t) is a multivariate time-series with *N* state variables. Formation of RP requires the creation of a *T*-by-*T* binary matrix *R* based on ESR. Its element $R_{i,j}$ is determined by the Ref. [18]:

(17) $$R_{i,j}=\Theta \left(\tau_{RP}-d\left(x\left[i\right],x\left[j\right]\right)\right),$$

for 1≤i,j≤T, d(·,·) is a Euclidean-based dissimilarity measure, Θ(·) is the Heaviside function, and $\tau_{RP}>0$ is a threshold for detecting recurrences. For a more detailed description, we direct the readers to the Ref. [18].

After binary matrix *R* has been generated, we proceed to prepare the histograms that count the number of diagonal lines with length *l* and vertical lines with length *v* as follows

(18) $$P\left(l\right)=\sum_{i,j=1}^{T-l}\left(1-R_{i-1,j-1}\right)\left(1-R_{i+l,j+l}\right)\prod_{k=0}^{l-1}R_{i+k,j+k}$$

(19) $$P\left(v\right)=\sum_{i,j=1}^{T-v}\left(1-R_{i,j}\right)\left(1-R_{i,j+v}\right)\prod_{k=0}^{v-1}R_{i,j+k}$$

Based on the histograms, we then measure the maximum diagonal line length, $L_{MAX}$ as follows

(20) $$L_{MAX}=max{\left\{l_{i}\right\}}_{i=1}^{N_{l}}$$

where $l_{i}$ is the length of *i*-th diagonal line, $N_{l}$ is the total number of diagonal lines, and $1\leq L_{MAX}\leq \sqrt{2}T$. This measure is a state-of-the-art stability measure of ESN reservoirs suggested by the Ref. [18]. It quantifies the degree of reservoir stability based on the diagonal lines in RPs of ESRs: the higher the $L_{MAX}$, the more stable the reservoir. In the following process, we will start tuning input scaling and spectral radius subject to this reservoir stability criterion.

When tuning input scaling and spectral radius for reservoir stability, primarily, we must first ensure the ESP condition for ESR existence. Hence, we split the hyperparameter tuning process into two stages. In the first stage, considering the previous literature that suggested that we must set $S_{R}$ close to but less than 1 to ensure the ESP [9], we hence fix $S_{R}$ at a constant boundary value of 0.99 [10]. Meanwhile, we iteratively adjust the values of $I_{S}$ and measure its corresponding degree of stability measured by $L_{MAX}$. Observing the response of $L_{MAX}$ for different values of $I_{S}$, the value of the highest $L_{MAX}$ indicates the optimal configuration for a stable self-organizing reservoir. In the second stage, applying the empirically tuned input scaling, we fix input scaling and iteratively vary the values of $S_{R}$ and measure its corresponding degree of stability indicated by $L_{MAX}$. In particular, we are interested in investigating the stability of the self-organizing reservoir when violating ESP, which is setting $S_{R}$ to be greater than unity. Similarly, the value of the highest $L_{MAX}$ indicates the optimal configuration of $S_{R}$ for a stable self-organizing reservoir. The proposed two-stage hyperparameter tuning approach guarantees optimal configuration of $I_{S}$ and $S_{R}$ for stable reservoir design and the satisfaction of ESP. Hence, optimized self-organizing reservoirs are generated.

Besides, we further measure additional RQA metrics and perform several explainability analyses by visualizing the dynamical behavior of the self-organizing reservoir in the context of the HAR problem. In this work, we focus on three dynamical behaviors: laminarity, time dependence and chaoticity [18]. Laminarity, LAM∈[0,1] quantifies the existence of laminar phase in a reservoir as follows

(21) $$LAM=\frac{\sum_{v=v_{min}}^{T}vP\left(v\right)}{\sum_{v=1}^{T}vP\left(v\right)}$$

where $v_{m}in$ is the minimal vertical line length used as a threshold. The higher the value of LAM, the more significant a reservoir poses laminar phase, and its echo state changes very slowly for several adjacent time steps. In RP of a reservoir, the occurrence of large black rectangles exhibits laminar phase.

Determinism level, DET∈[0,1] quantifies the existence of time dependence in a reservoir as follows

(22) $$DET=\frac{\sum_{l=l_{min}}^{T}lP\left(l\right)}{\sum_{l=1}^{T}lP\left(l\right)}$$

where $l_{min}$ is the minimal diagonal line length used as a threshold. The higher the value of DET, the more significant a reservoir poses time dependence. Non-uniformly distributed RPs indicates the presence of time dependence.

Recurrence rate, RR quantifies the chaoticity in a reservoir as follows

(23) $$RR=\frac{1}{T^{2}}\sum_{i,j=1}^{T}R_{i,j}$$

It measures the recurrences density, and a low value of RR indicates the existence of chaoticity in the reservoir. In RP, the occurrence of short and erratic diagonal lines indicates chaoticity.

**Figure 3:**
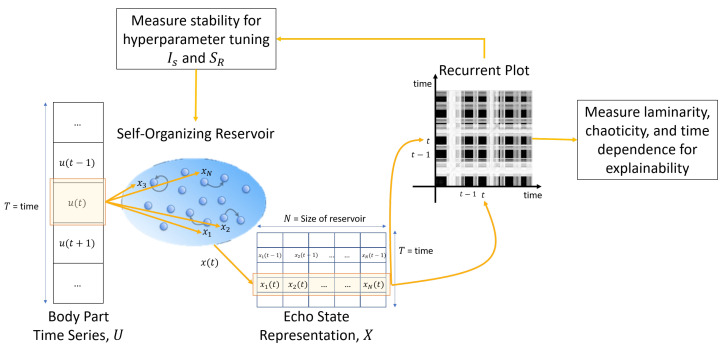
Generating RP for hyperparameter tuning and explainability analysis using RQA measure. u(t) is the action input at instant *t*. Projecting u(t) onto the self-organizing reservoir with *N* neurons generates an echo state x(t).

In HAR, human actions are considered as multivariate time-series data. We hypothesize that projecting human action data onto the self-organizing reservoir generates ESR showing significant laminarity and time dependence dynamics in RPs. Moreover, a stable reservoir offers less chaoticity. Figure 3 summarizes the workflow to generate RP for hyperparameter tuning and explainability analysis using RQA measures.

### 3.3. Self-Organizing Convolutional Echo State Network (SO-ConvESN)

**Figure 4:**
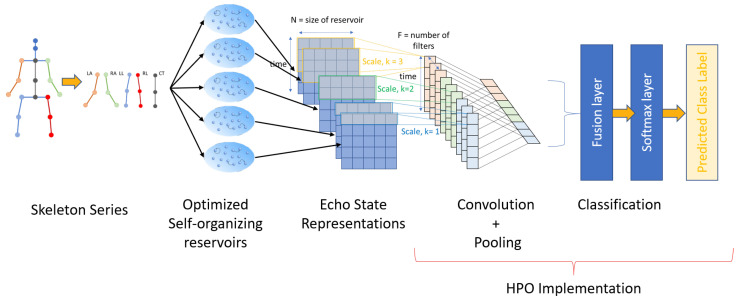
Overview architecture of SO-ConvESN. Optimized self-organizing reservoirs are generated by SORN-E and cascaded in multi-scale CNN with three time-scales, three filters and five channels for human action recognition. HPO implementation is solely conducted for the CNN stage.

In this section, we present the applicability and feasibility of the proposed SORN-E. Following the direction of ConvESN, the proposed SORN-E is cascaded with a simple CNN to yield a SO-ConvESN for the HAR task. SORN-E is composed of the optimized self-organizing reservoirs and echo state representations of actions series. CNN consists of convolutional-pooling layers and a classification stage. Figure 4 depicts the general framework of the SO-ConvESN for human action recognition. For ease of visualization, we display the SO-ConvESN with only three filters.

In the context of HAR, each frame of the skeleton series is first split into five individual channels. Each channel corresponds to each 3D-skeleton joint coordinate trajectories of five body parts; left arm (LA), right arm (RA), central trunk (CT), left leg (LL) and right leg (RL).

Each frame of a body part, u(t), is treated as multivariate time-series in the SORN-E stage. For each time instant, t∈[0,T−1], u(t) is mapped into the respective optimized self-organizing reservoir, and echo states are extracted according to Equation (15) and collected into the ESR matrix as in Equation (16). ESR matrix is the complete representation of the *T*-length input skeleton series. CNN then extracts the discriminative multiscale temporal features from ESR and determines the action class.

**Figure 5:**
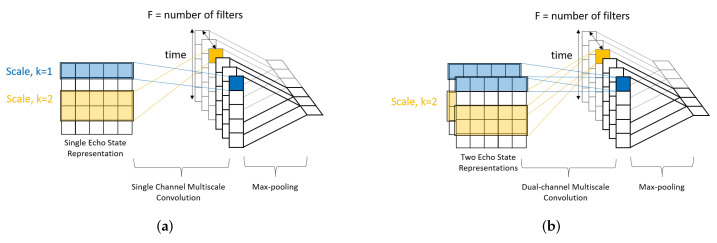
Multiscale convolution process extracts temporal features from ESR. (**a**) Single channel convolution is used for CT channel; (**b**) Dual-Channel Convolution is used for LA and RA as well as LL and RL channels.

In the CNN stage, the process starts with the multiscale convolution operations along the rows of the ESR matrix. In other words, the convolutions are along the direction of time. Multiple convolution filters with bias terms extract temporal features according to each time scale, *k*. Figure 4 depicts three examples with k=1, 2, and 3, and each time scale uses F=3 filters. Hence, a convolution filter is of size k×N. The central trunk is considered as general multidimensional time-series without any correlation with another channel. However, the spatial correlation between the skeleton joints of left channel and right channel of the arms as well as the left channel and right channel of the legs are highly associated when a subject is performing an action. Hence, they are considered as multivariate time-series with spatial correlation. The single channel multiscale convolution process is not suitable to tackle such scenario. Following the baseline ConvESN, we separately apply dual-channel multiscale convolution operation on the ESRs of left channel and right channel of the arms as well the ESRs of the left channel and right channel of the legs. Figure 5a,b illustrate the single channel and dual-channel multiscale convolution process with F=3 and k=1,2.

Similar to ConvESN, we use max-over-time pooling [55] to extract the maximum from the feature maps, which selects the most discriminative local feature regardless of which time step it locates. Max-over-time pooling maintains multiscale temporal invariance. The pooled features are collected into one scalar and then concatenated as one single vector. The output vector of the pooling layer enters a fully connected layer followed by the softmax layer for classification. Assuming $C_{s}$ represents the *s*-th class of actions, the softmax layer yields conditional distribution $P(C_{s}|u)$ over action classes.

After defining the architecture of the proposed SO-ConvESN, deploying for the HAR task requires prior weights training of CNN to be performed. However, the training of a CNN requires the specifications of multiple primary hyperparameters such as number of filters, learning rate, batch size, kernel size, optimizers, activation functions, and the number of epochs. Some of these hyperparameters greatly influence the network performance, and finding the optimal configurations can be viewed as a search problem. Based on preliminary experiments, hand tuning the optimizer and activation function do not show significant impact on the network performance. Therefore, we predefined rectified linear unit (ReLU) [56] as the non-linear activation function in CNN because of its computational efficiency and fast convergence. Meanwhile, the convolution kernel weights are optimized by Adam [14] which is essentially designed to adjust the learning rate adaptively for deep neural network training. But the network is still highly sensitive to the selection of initial learning rate. The cross-entropy error function is used as the training loss during weights optimization as for HAR is a classification problem.

On the other hand, this work explores the optimal configuration of three crucial hyperparameters of CNN: the learning rate, number of filters, and batch size, by applying HPO algorithms. We focus on exploring the feasibility of three HPO algorithms: BO, ASHA, and PBT, for the sake of investigating and selecting the most well-suited method to configure these CNN hyperparameters. We refer the readers to the works of [20,21,22] for further detailed descriptions of BO, ASHA, and PBT, respectively.

During the HPO of CNN in SO-ConvESN, it accepts ESR generated from the projection of action series onto the self-organizing reservoir according to Equations (15) and (16) as inputs and returns the recognition accuracy as numerical output as follows

(24) $$Accuracy=\frac{Number\;of\;correctly\;recognized\;samples}{Total\;number\;of\;samples}$$

**Figure 6:**
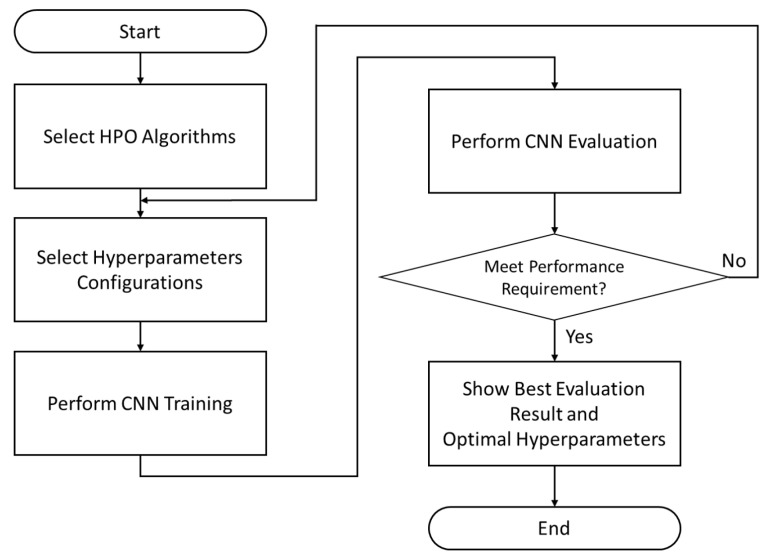
A general search loop of HPO implementation in CNN stage of SO-ConvESN. Every optimization run loop will train a CNN based on the selected hyperparameters and evaluate the recognition accuracy based on the training and validation datasets. best-performing configuration and the corresponding accuracy are the outcomes of the search loop.

HPO aims to find the hyperparameters that maximize the HAR accuracy efficiently. Figure 6 summarizes the general flow of the search loop process.

In general, an HPO algorithm first selects the possible valid configurations of hyperparameters from the search space. It then trains the CNN using the training dataset and evaluates the recognition accuracy using the validation dataset in every optimization run. The ultimate goal of HPO is to determine a set of best-performing hyperparameters. The search space of the CNN hyperparameters is problem-dependent. The detailed configuration of the search space is empirically demonstrated during simulation experiments.

## 4. Results and Discussion

Intending to demonstrate the capability of the SORN-E in generating stable self-organizing reservoirs and investigate the potential of HPO algorithms for optimizing the recognition performance of the SO-ConvESN, several simulation experiments are conducted using three openly available skeleton-based action recognition benchmark datasets: MSR-Action 3D (MSRA3D) [57], Florence3D-Action (Florence3D) [58], and AHA3D [59]. Each dataset includes a different collection of actions and gestures. AHA3D dataset is merely used for the deployment of the proposed framework for rehabilitation application.

We divide the simulation experiments into several parts. In the first experiments, we present the results in evaluating the feasibility and applicability of the proposed SORN-E to generate stable self-organizing reservoirs. Applying RQA techniques helps to investigate the sensitivity of two crucial hyperparameters of the reservoir and hence select the optimal configurations that ensure stability and ESP. We also aim to achieve more insight into the dynamics and reveal the explainability of the self-organizing reservoir by visualizing the ESRs via RPs and heatmaps.

In addition, the proposed SORN-E is cascaded with a CNN to deal with 3D-skeleton-based action recognition tasks. In the second experiment, we demonstrate the applicability and showcasing of the HPO algorithms in hyperparameter tuning for the CNN of SO-ConvESN. Results of different HPO algorithms are compared and investigated to determine the well-suited HPO algorithm for optimizing the CNN of SO-ConvESN. The last part shows the performance comparison with state-of-the-art approaches and the deployment of SO-ConvESN for HAR application.

### 4.1. Introduction of Benchmark Datasets

As mentioned before, we adopt three public skeleton-based action recognition benchmark datasets. The first dataset is MSRA3D, composed of 567 sequences with 23,797 skeleton frames recorded at 15 fps with each action performed by ten different subjects 2 or 3 times. It is one of the most famous HAR benchmark datasets used by researchers, which employed Kinect-like sensor to acquire 20 skeleton joints for 20 different activities: high-arm wave, horizontal arm wave, hammer, hand catch, forward punch, high throw, draw X, draw tick, draw a circle, hand clap, two-hand wave, side boxing, bend, forward kick, side kick, jogging, tennis swing, tennis serve, golf swing, and pick-up and throw. Instead of discarding some of the skeleton frames with excessive noise which are missing or corrupted, like in the Refs. [60,61], this work uses the entire dataset intending to assess the noise handling capability of the proposed self-organizing learning to generate reservoirs.

The second dataset is Florence3D, composed of 215 sequences with each action performed by ten different subjects two or three times. It used a Kinect sensor to record 15 skeleton joints for nine various activities: wave, drink from a bottle, answer phone, clap, tight lace, sit down, stand up, read watch, and bow. In this dataset, the same action is performed with both left and right hands, and it consists of actions with high similarities, such as drinking from a bottle and answering the phone. This high intraclass variation makes the recognition task more challenging.

The last dataset is AHA3D, composed of 79 different 3D skeletal videos with 171,753 skeleton frames, with each exercise action being performed one to three times by 21 subjects. It used a Kinect sensor to capture 21 skeleton joints for four different standard fitness exercises: 30 s chair stand, 8 ft up and go, two-minute step test, and unipedal stance. Similar to Florence3D, high intraclass variation makes the recognition task more challenging. The same exercises were performed by the mixture of 11 young subjects and ten elderly subjects. Five of the subjects were male, and 16 were female.

Adopting these three datasets during the reservoir design, explainability analysis, and the HPO of CNN, the proposed SORN-E and SO-ConvESN can be efficiently and empirically evaluated and compared with the state-of-the-art HAR approaches.

### 4.2. Implementation Details

To begin with, we performed preprocessing of all the raw skeletal sequences in all datasets before using the datasets to train and evaluate the SORN-E and SO-ConvESN. Since the origin references of the raw skeleton joints in a given sequence are different from each other, we first transformed the raw skeleton joints into a normalized coordinate system. For each skeleton sequence, we determined the average of the center, left, and right hip joints and set the origin of every frame in the skeleton sequence to this average point. Then, we applied a Savitzky–Golay smoothing filter [62] to smoothen the normalized skeleton sequences containing different smoothness levels. To ensure each sample to have the same length trajectories, we padded the skeleton sequences up to the maximum length with zeros.

We adopted different training and validation protocols according to the standard proposed in each benchmark dataset to support unbiased comparison with the state-of-the-art performances. In the MSRA3D experiment, we implemented the standard validation protocols as in the Ref. [57]. We created three sets of training and validation configurations where samples from half of the subjects were used as training dataset, and the balance was used as a validation dataset. Meanwhile, in the Florence3D experiment, ten-fold cross-validation protocols as used by the Ref. [58] were adopted for the training and validation process. As for AHA3D experiment, we followed the protocol proposed in the Ref. [59]. We split the available 79 skeletal videos into 39 videos for training, 20 videos for validation, and 20 videos for testing. The performance accuracy was measured and averaged over 100 runs. During performance evaluation and comparison, we employed accuracy as in Equation (24) as the metric for the context of the HAR problem. Better performance is indicated by a higher value of the computed accuracy.

In term of the implementation of SORN-E, we conducted preliminarily experiment of SORN learning to determine the number of self-organizing reservoir neurons. Even though SORN learning adaptively grows the network size, this work used the number of neurons as stopping criterion to fix the size of the reservoir. Whereas for the implementation of RPs and RQA for reservoir explainability, we adjusted the threshold $\tau_{RP}$ and ensure $\tau_{RP}>0$ for detecting recurrences. For SORN reservoir, the configurations of input scaling, $I_{S}$ and spectral radius, $S_{R}$ were tuned by RPs and RQA based techniques and later were fixed to optimal values of 0.1 (0.09 for Florence3D) and 0.99, respectively to ensure stability and ESP for the deployment into SO-ConvESN.

In term of the implementation of HPO algorithms for CNN in SO-ConvESN, we configured the same search space of number of filters, learning rate, batch size for BO, ASHA, and PBT according to the benchmark dataset used. We essentially consolidated the optimization efforts on the learning rate with continuous range. We fixed the convolutional kernel size as 2, 3, and 4 for multiscale feature extraction [10]. PBT is no well-suited to tune hyperparameters that vary the network architecture. Perturbing these hyperparameters may void the weights inherited from best-performing generation. Hence, we fixed the number of filters to adapt PBT during HPO process. Table 1, Table 2 and Table 3 show the search space setups of the MSRA3D, Florence3D, and AHA3D datasets, respectively.

We ran BO, ASHA, and PBT in this experiment with the same respective configuration across all datasets. The hyperparameters of the CNN were optimized with BO algorithm with the expected improvement (EI) as the acquisition function. This process was repeated for 5 iterations for each run with a total of 25 independent runs. For ASHA, we set reduction factor, η to 4, minimum resource, *r* to 1, maximum resource, *R* to 64 and minimum early-stopping rate, *s* to 0. For PBT, we ran HPO with a population size of 10 with five generations. We implemented truncation selection for the exploit stage. During exploration, inherited learning rate were perturbed by either 0.8 or 1.2.

All experimental simulations were conducted in an Intel Core i5-9300H, 2.40 GHz CPU. The algorithms were developed and executed in the Python platform. The time elapsed to produce a self-organizing reservoir by the SORN learning consumed average of 1.6 ms per frame, 1.0 ms per frame, and 3.5 ms per frame for MSRA3D, Florence3D, and AHA3D datasets, respectively.

### 4.3. Effects of Hyperparameters in Self-Organizing Reservoir Network (SORN)

The effects of the hyperparameters configuration were examined by two benchmark datasets: MSRA3D and Florence3D. The results were obtained by empirically experimenting with different hand tuned settings over multiple SORN learning sessions.

As mentioned earlier, the proposed SORN expands the network size adaptively during the learning process. In order to define the size of the self-organizing reservoir, this work used the user-defined maximum number of neurons as stopping criterion. In addition, the learning cycle was set as 10% of the max number of nodes. It implies that the pruning process will be regularly performed for every iteration reaching 10% of the predefined maximum number of nodes. Preliminary experiments showed that as long as the number of reservoir neurons is set to at least three times the input dimension, the reservoir possesses sufficient complexity to capture and hold the information of the input samples. Considering the length of one body part with three skeleton joints, each of them has three coordinate values, contributing to an input length of nine features. Hence, throughout the simulations, we fixed the number of neurons at 36 for MSRA3D. For Florence3D, we set the number of neurons at 27. For AHA3D, we set the number of neurons at 50. Arbitrarily setting the learning rate showed no noticeable impact on the performance. We fixed it at 0.5 to a moderate learning rate during the update of winner nodes.

**Figure 7:**
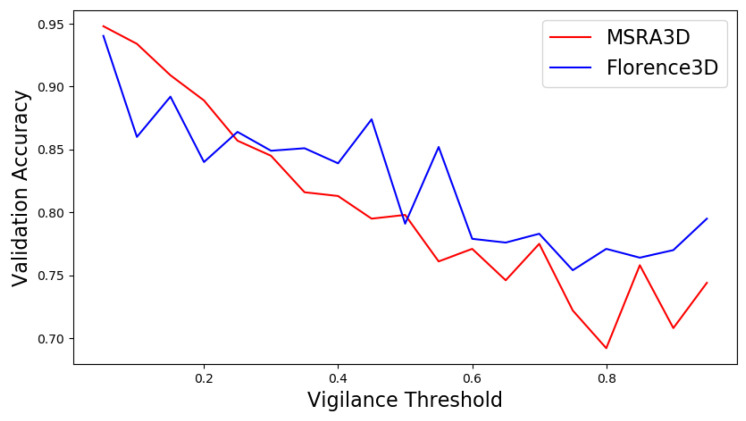
The graphs depict the impact of tuning the vigilance threshold from 0.05 to 0.95 on the performance of SO-ConvESN in HAR task based on MSRA3D dataset (Red Solid line) and Florence3D dataset (Blue Solid line). In both models, when the vigilance threshold increases, validation accuracy drops.

In terms of the vigilance threshold and reservoir perturbation of SORN, we conducted different training sessions by using a range of values and insert the generated self-organizing reservoirs into SO-ConvESN for HAR tasks using only MSRA3D and Florence3D. We manually tuned the vigilance threshold for a range from 0.05 to 0.95, and the initial noise distribution scales at 0, 0.1, 0.01, and 0.001 to observe the SORN in performing clustering for HAR task. As shown in Figure 7, the results show that configuring the vigilance threshold to 0.05 produced optimal clustering by SORN. Compared to other configurations, setting the vigilance threshold to a low value resulted in the highest validation accuracy in both MSRA3D and Florence3D datasets. Whereas configuring the noise perturbation at 0.1, SO-ConvESN outperformed the noise-less version, and the results showed no noticeable impact for the magnitudes set at 0.01 and 0.001.

The results suggest that setting the vigilance threshold at low value is essentially to produce self-organizing clustered topology of nodes with high-granularity. These clusters at a significantly reduced dimensionality can efficiently represent the topological properties of the complete set of input skeleton joint samples. Besides, implementing a tunable noise perturbation as in Equation (1) to control the amount of the initial noise to be added to the input sample before SORN learning offers adjustment for fast convergence clustering [54]. Configuring the noise perturbation to 0.1 enabled SORN-generated self-organizing reservoirs unlocked better performance of the SO-ConvESN.

### 4.4. RQA-Based Hyperparameter Tuning for Self-Organizing Reservoirs

These additional experiments demonstrate the applicability of the proposed SORN-E to adjust the spectral radius, $S_{R}$ and the input scaling, $I_{S}$ in generating reservoirs that are stable yet satisfied ESP. We conducted the simulation experiments by using both MSRA3D and Florence3D datasets. As described previously, we divided the hyperparameter tuning process into two stages.

**Figure 8:**
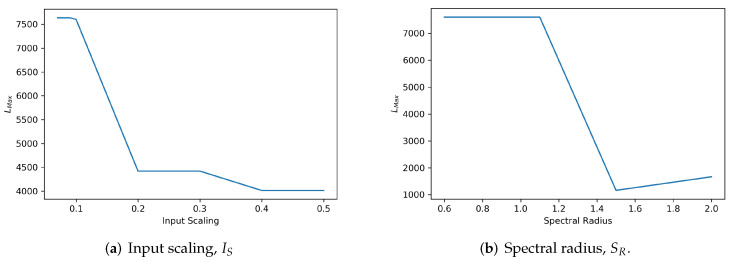
Results on the MSRA3D dataset. Measured reservoir stability, $L_{MAX}$ against different hyperparameter settings.

**Figure 9:**
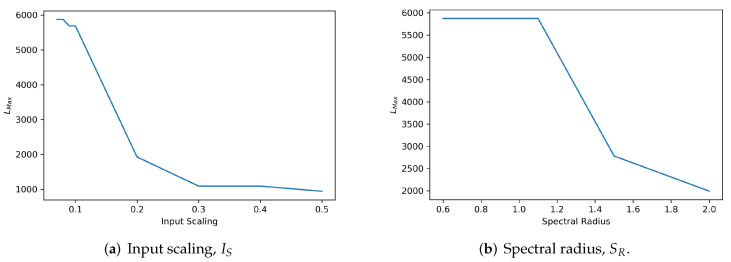
Results on the Florence3D dataset. Measured reservoir stability, $L_{MAX}$ against different hyperparameter settings.

In the first stage, we fixed the spectral radius, $S_{R}$ at a constant boundary value of 0.99 on the basis of the previous study showing that this value ensures the ESP [9,10]. We then generated ESR and measured $L_{MAX}$ that indicates the degree of quantified reservoir stability based on Equation (20) against the variation of the input scaling, $I_{S}$. A higher value of the $L_{MAX}$ represents a more stable configuration of input scaling. We hand tuned the input scaling for a range from 0.07 to 0.5 and measure the respective $L_{MAX}$. We repeated the measurements using MSRA3D and Florence3D datasets. The measured values of $L_{MAX}$ against the different input scaling settings are depicted in Figure 8a and Figure 9a, respectively. Setting input scaling at a range less than or equal to 0.1 for the MSRA3D dataset and less than or equal to 0.09 for the Florence3D dataset shows optimal stability in which $L_{MAX}$ stays at the highest value.

In the second stage, we applied the optimal value determined in the previous step and fixed the input scaling. We then repeated the measurement of $L_{MAX}$ against the adjustment of the spectral radius, $S_{R}$. In particular, we investigated the stability of the self-organizing reservoir when violating ESP, which is setting $S_{R}$ to be greater than unity. Similarly, the value of the highest $L_{MAX}$ indicates the optimal configuration of $S_{R}$ for a stable self-organizing reservoir. The measured values of $L_{MAX}$ against the different spectral radius settings for MSRA3D and Florence3D datasets are depicted in Figure 8b and Figure 9b, respectively. Interestingly, setting $S_{R}$ to be slightly greater than unity to violate ESP, self-organizing reservoir stays stable but only sustains up to a certain extend. In both MSRA3D and Florence3D scenarios, the results prove that setting spectral radius less than unity guarantees ESP and ensures stability.

We have applied the proposed SORN-E in generating stable self-organizing reservoirs for all five channels in correspondence to five body parts of the skeleton joints. For simplicity, this section solely showed the results of a single channel self-organizing reservoir. Based on the findings of this experiment, both chosen spectral radius and input scaling affects the stability of the reservoirs. The proposed SORN-E is feasible and applicable to guarantee optimal configuration of $I_{S}$ and $S_{R}$ for stable reservoir and the satisfaction of ESP. We demonstrated the applicability and showcasing of the RQA technique in designing stable self-organizing reservoir. Explainability of the $L_{MAX}$ metric essentially quantifies the reservoir stability and is one of the helpful tools to guide the reservoir design, especially tuning the hyperparameters. In a nutshell, the proposed SORN-E is capable of generating optimized self-organizing reservoirs that are stable and yet fulfilled ESP.

### 4.5. Comparison between the Self-Organizing Reservoirs and Randomly Initialized Reservoirs Based on Explainability

In the following, we considered that the proposed SORN-E had generated stable self-organizing reservoirs as described previously. We applied the same hyperparameter configuration to generate randomly initialized reservoirs. We compared the self-organizing reservoirs and randomly initialized reservoirs by quantifying the reservoir dynamics via RQA metrics and the visualization of the RPs of ESRs.

**Figure 10:**
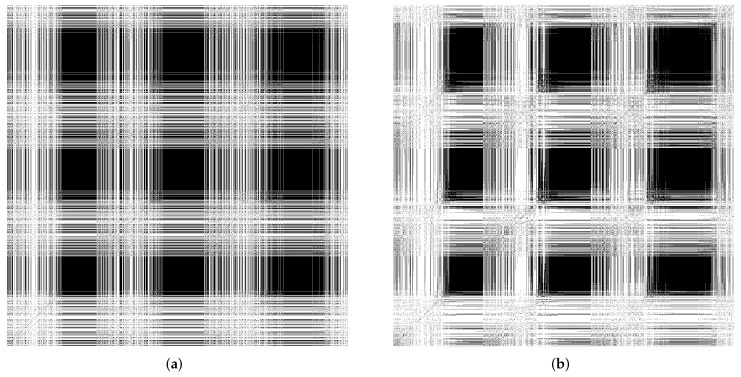
Recurrence plots using MSRA3D dataset and setting optimal $S_{R}$ at 0.99 and $I_{S}$ at 0.1. (**a**) Self-organizing reservoir with RQA metrics: $L_{MAX}$ = 7601, LAM = 0.999905, DET = 0.999976, RR = 0.999052; (**b**) Randomly initialized reservoir with RQA metrics: $L_{MAX}$ = 4419, LAM = 0.989145, DET = 0.985305, RR = 0.931044.

**Figure 11:**
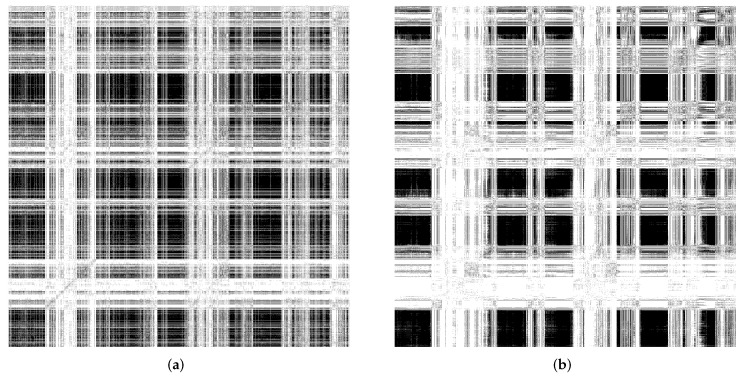
Recurrence plots using Florence3D dataset and setting optimal $S_{R}$ at 0.99 and $I_{S}$ at 0.09. (**a**) Self-organizing reservoir with RQA metrics: $L_{MAX}$ = 5874, LAM = 0.999980, DET = 0.999999, RR = 0.998964; (**b**) Randomly initialized with RQA metrics: $L_{MAX}$ = 5075, LAM = 0.998944, DET = 0.999626, RR = 0.992221.

We measured RQA metrics and visualized the RPs of ESRs produced by the reservoirs using the MSRA3D and Florence datasets. We aimed to reveal the explainability of the reservoir dynamics for the context of HAR. We focused on three essential classes of reservoir dynamics: laminarity, time dependence, and chaoticity. Figure 10a,b depict the recurrent plots generated using the MSRA3D dataset. Figure 11a,b depict the recurrent plots generated using the Florence3D dataset. We had discovered similar findings of all five channels of action sequences. For visualization simplicity, we merely included the RPs of a single channel.

Comparing the stability measure via $L_{MAX}$, self-organizing reservoir scores higher values at 7601 and 5874 in MSRA3D and Florence3D datasets, respectively as compared to randomly initialized reservoir. In terms of laminarity, the RPs of both self-organizing reservoir and randomly initialized reservoir shows that echo state varies very gradually over a number of adjacent time steps which can be observed by the presence of large black rectangles. In both MSRA3D and Florence3D, RPs visualizes the existence of laminarity. Comparing the measure LAM, self-organizing reservoir scores higher values at 0.999905 and 0.999980 in MSRA3D and Florence3D dataset, respectively as compared to randomly initialized reservoir. Besides, the RPs are non-uniformly distributed that visualize the time dependence dynamics. It means both reservoirs have captured the correlation of the action sequences. Self-organizing reservoir has superiority in capturing time dependency which can be observed by the measured DET which is closer to 1 as compared to randomly initialized reservoir in both datasets. The measured RR of self-organizing is also closer to 1 which indicates lesser chaoticity than the randomly initialized reservoir.

Based on the experimental findings, the proposed SORN-E has successfully generated self-organizing reservoirs that are more stable, pose a higher laminarity phase, higher time dependence, and lesser chaoticity. The self-organizing reservoirs have significantly preserved these essential signature dynamics for HAR, which can be understood via the explanatory information extracted from ESRs. Applying SORN-E for deterministic initialization on the ESN’s weight not only ensures stability and ESP, but self-organization also ensures the action sequences’ dynamics are better reflected and captured by the reservoir neuron activations. The SORN-E can be considered one feasible and biologically plausible self-organizing reservoir design approach, notably, encode human actions’ temporal feature for HAR.

### 4.6. Comparison between the Self-Organizing Reservoirs and Randomly Initialized Reservoirs Based on Reproducibility

**Figure 12:**
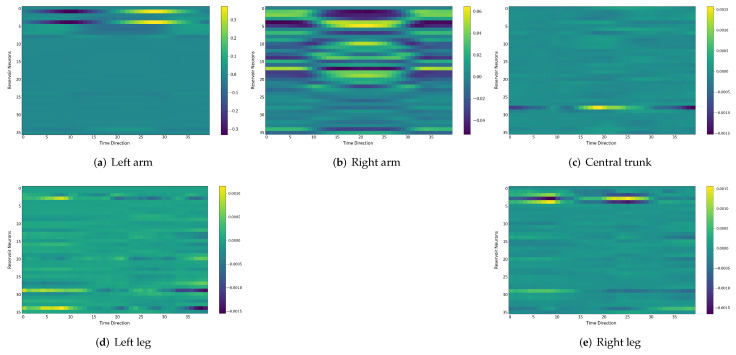
Visualization of ESRs for an action sequence of a person performing two-hand waving with 40 frames that was projected onto the self-organizing reservoirs with 36 neurons generated by SORN-E. Vertical axis indicates the number of reservoir neurons and horizontal axis indicates the time frames. The results are produced by projecting the same action time-series onto self-organizing reservoirs to produce ESRs for three different trial runs. Self-organizing reservoirs ensure deterministic initialization of the reservoir weights for reproducibility.

**Figure 13:**
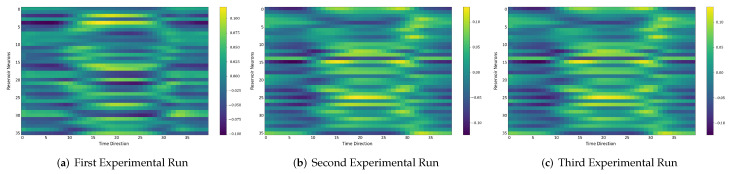
Visualization of ESRs of randomly initialized reservoir with 36 neurons for left arm trajectories of a person performing two-hand waving.

**Figure 14:**
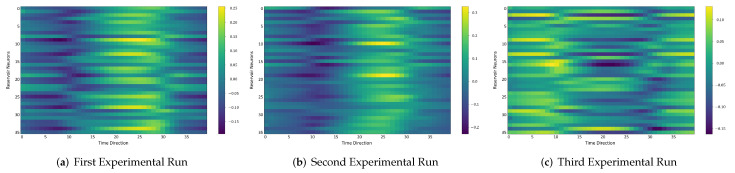
Visualization of ESRs of randomly initialized reservoir with 36 neurons for right arm trajectories of a person performing two-hand waving.

**Figure 15:**
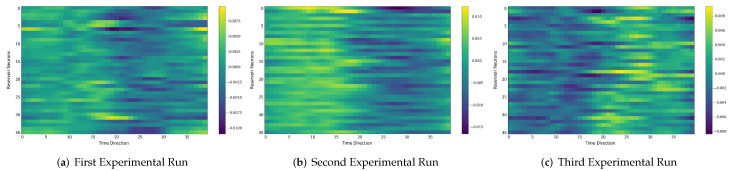
Visualization of ESRs of randomly initialized reservoir with 36 neurons for central trunk trajectories of a person performing two-hand waving.

**Figure 16:**
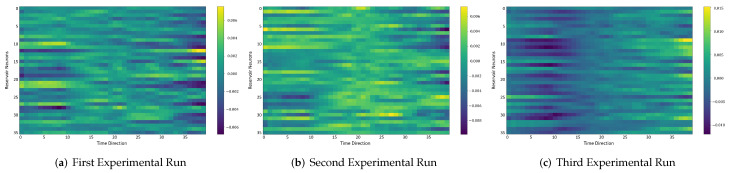
Visualization of ESRs of randomly initialized reservoir with 36 neurons for left leg trajectories of a person performing two-hand waving.

**Figure 17:**
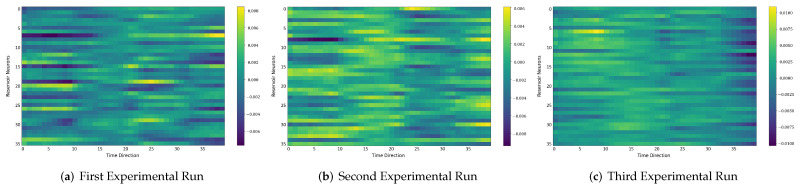
Visualization of ESRs of randomly initialized reservoir with 36 neurons for right leg trajectories of a person performing two-hand waving.

In the following, we additionally presented the visualization of ESRs using the MSRA3D dataset. We aimed to investigate the reproducibility of the self-organizing reservoirs generated by SORN-E. For simplicity, we visualized the heatmaps for ESRs of the left arm, right arm, central trunk, left leg, and right leg trajectories of a person performing two-hand waving. We projected this same action input sequence of 40 frames onto the ESN reservoirs to repeat three different trial runs independently. Figure 12, Figure 13, Figure 14, Figure 15, Figure 16 and Figure 17 show the heatmaps of the ESRs for self-organizing reservoirs and randomly initialized reservoirs, respectively.

The visualizations of ESRs show that a self-organizing reservoir reproduces the same heat maps for the identical action sequences in different runs. Conversely, randomly initialized reservoirs produce different ESRs in separate runs. The generated ESRs do not show regular neuron activations behaviors. It is interesting to note that the neuron activations of the self-organizing reservoir along the time length are highly specific. Only a particular group of signature neurons are activated at a time. This group of neurons relevant to a particular part of the body exhibit higher activations at any one time. Whereas for randomly initialized reservoirs, the behaviors are unpredictable and act stochastically. Randomly initialized reservoir failed to reproduce same ESRs in different runs. Moreover, the activations of the neurons do not show any sign of body part relevancy. In other words, randomly initialized reservoirs hardly achieve reproducibility even using the same set of hyperparameter configurations.

Based on the findings of the visualization experiments, SORN-E generates self-organizing reservoirs that preserve the reproducibility for the same input action sequence due to its deterministic initialization of the ESN’s reservoirs. Additionally, a particular body part explicitly activates a specific group of neurons during an action. This finding shows that neurons of the self-organizing reservoirs could be task-specific neurons. This result may also justify that SORN-E produces self-organizing reservoirs that follow the biological mechanism of adaptation of neuron excitability.

### 4.7. Optimizing the Performance of SO-ConvESN

**Figure 18:**
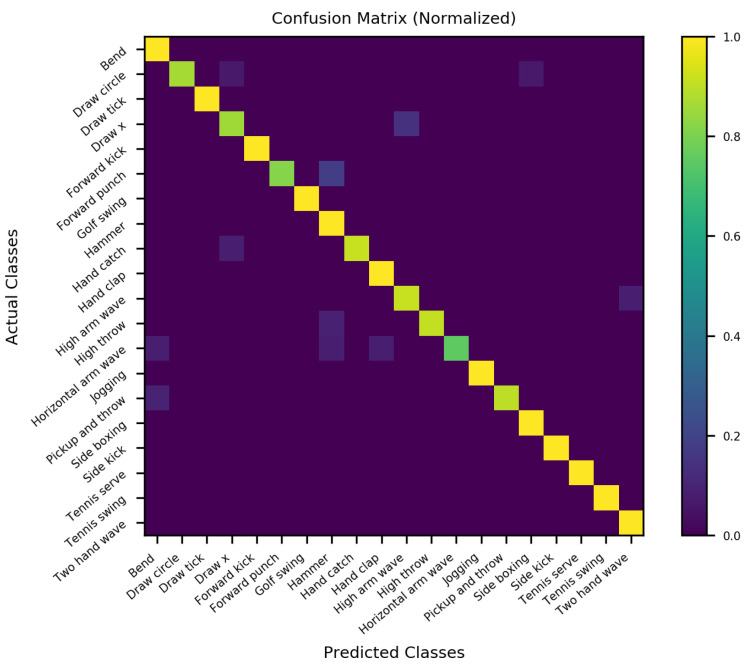
The normalized confusion matrix for SO-ConvESN-ASHA on MSRA3D dataset.

**Figure 19:**
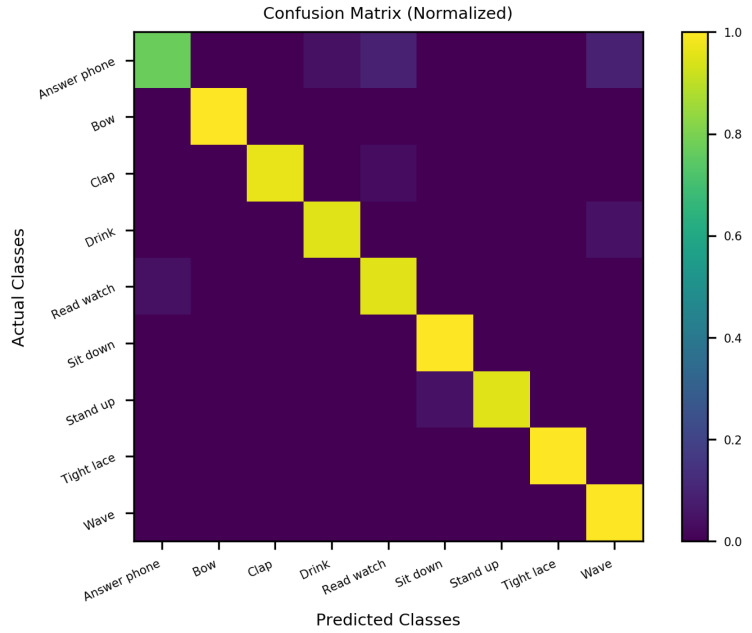
The normalized confusion matrix for SO-ConvESN-ASHA on Florence3D-Action dataset.

As highlighted earlier, we cascaded the optimal self-organizing reservoir generated by SORN-E with a simple CNN to yield the SO-ConvESN. In this section, to ensure optimal HAR performance, we implemented BO, AHSA, and PBT algorithms to optimize the HAR accuracy and compared the results of different HPO techniques for the CNN stage that performs the multiscale convolutional process in SO-ConvESN using the MSRA3D and Florence datasets. We also included the baseline SO-ConvESN hand-tuned manually to explore the optimal number of kernels in CNN by specifying the scales from 16 to 256 and the learning rate from 0.001 and 0.003. Table 4 depicts the HAR results achieved by the implemented HPO algorithms. Figure 18 and Figure 19 show the normalized confusion matrices of the recognition accuracy achieved by SO-ConvESN-ASHA for MSRA3D dataset and Florence3D dataset, respectively.

We demonstrate the applicability and showcasing of the HPO algorithms in hyperparameter tuning of CNN in SO-ConvESN to justify that HPO is required to further improve the recognition accuracy in the baseline SO-ConvESN. Based on the experimental observations, all implemented HPO algorithms have improved the recognition accuracy of SO-ConvESN. In MSRA3D models, as compared to the baseline performance, BO improved the accuracy by 0.63%, ASHA achieved improvement of 0.93%, and PBT showed 0.30% better accuracy. Whereas in the Florence3D models, BO showed 4.83% improvement, ASHA achieved 7.10% improved accuracy, and PBT improved the performance by 2.47%. The ASHA significantly showed the best accuracy improvement in optimizing SO-ConvESN in both datasets, whereas BO outperformed PBT in both experiments.

The results demonstrate the effectiveness of ASHA in optimizing the proposed SO-ConvESN for HAR. The inherently sequential optimization of BO makes this method unsuitable for optimizing CNN in SO-ConvESN. For PBT, it could be due to the nature of hyperparameters that variances in the network architecture needs to be fixed. Manual setting of the number of filters in this experiment may degrade the optimization effect when applying PBT. It is also interesting to note that the recognition accuracy in MSRA3D experiments using either of the HPO algorithms exhibit a smaller factor of improvement as compared to Florence3D experiments. We inferred that including the noisy data from the MSRA3D dataset during SORN learning seems to be challenging for SO-ConvESN. We developed the SORN learning based on the ART model. It could be potentially sensitive to noise and makes the SORN-E unable to tolerate unmanageable, noisy MSRA3D data.

In the next section, we further compared the performances of the optimized SO-ConvESNs with the existing HAR approaches using the MSRA3D and Florence datasets. We abbreviated the SO-ConvESN optimized by BO, ASHA, and PBT as SO-ConvESN-BO, SO-ConvESN-ASHA, and SO-ConvESN-PBT, respectively. Table 5 and Table 6 tabulate the state-of-the-art HAR performance of cross-subject test on MSRA3D dataset and cross-validation on the Florence3D dataset.

For MSRA3D experiment, even our best-performing SO-ConvESN, that was optimized with ASHA, exhibited 95.09% overall accuracy. Compared to ConvESN, all of optimized SO-ConvESNs shows lower accuracy. The MSRA3D dataset could be a challenging dataset to SO-ConvESN. In particular, many actions are similar to each other, such as the “draw circle” action which was frequently misinterpreted as the “side boxing” action. Noisy data appearing in the MSRA3D dataset also make the noise-sensitive SORN-E fail to cope. However, considering SORN-E ensures stability and ESP in the self-organizing reservoir, reproducibility is more promising than the randomly initialized reservoir in ConvESN. For the Florence3D experiment, SO-ConvESNs outperformed ConvESN. Our best-performing SO-ConvESN, that was optimized with ASHA, exhibited 96.07% overall accuracy. SORN-E could be good at clustering data with low noise and a small number of action classes, as in Florence3D datasets.

Simulation experiments show that our proposed SO-ConvESN achieves competitive HAR performance with respect to the state-of-the-art approaches. The SORN-E considers self-organization inspired by cortex neuron adjustment mechanism and explainability for tuning ESN’s hyperparameters during the learning of reservoir weights ensures the stable self-organizing reservoirs capture the dynamics and topological properties of the input action sequences. The findings also show that the HPO algorithms are necessary to warrant improved recognition accuracy. The SO-ConvESN can be considered as one feasible and biologically plausible self-organizing reservoir design approach for the HAR problem.

### 4.8. Deployment of SO-ConvESN for Rehabilitation Application

Previous experimental studies have proven the applicability of the proposed SORN-E to generate stable self-organizing reservoirs, and yet satisfied ESP. Integrating SORN-E with a simple CNN has also been optimized via HPO algorithms to yield optimized SO-ConESN. Considering the outperformance of SO-ConvESN optimized by the ASHA, in this section, we demonstrate the deployment of the proposed approach for an assisted living-oriented performance assessment. We aimed to demonstrate the potential and usefulness of the SO-ConvESN-ASHA in an empirical application that recognizes physical fitness exercises for the elderly rehabilitation application using the AHA3D dataset.

This demonstration followed the proposed development framework as shown in Figure 1. First, the 79 skeletal videos of the AHA3D dataset were randomly split into 39 videos as the training set, 20 videos as the validation set, and 20 videos as the testing set. The training set was used for SORN learning and reservoir hyperparameter tuning. SORN-E generated node centroids and interconnectivity matrix and the optimal configuration of input scaling and spectral radius, which were then used to perform deterministic initialization of the recurrent weight and input weight in SO-ConvESN. The same training set and the previously selected 20 videos of the validation set were used to train and optimize SO-ConvESN using ASHA. Once trained, 20 videos of the testing set were used to evaluate the physical fitness exercise recognition performance of the SO-ConvESN-ASHA. This development and assessment process was iteratively conducted for 100 runs using 20 videos of testing set to recognize four fitness exercise actions: unipedal stance, 8 ft up and go, 30 s chair stand, and 2 min step.

**Figure 20:**
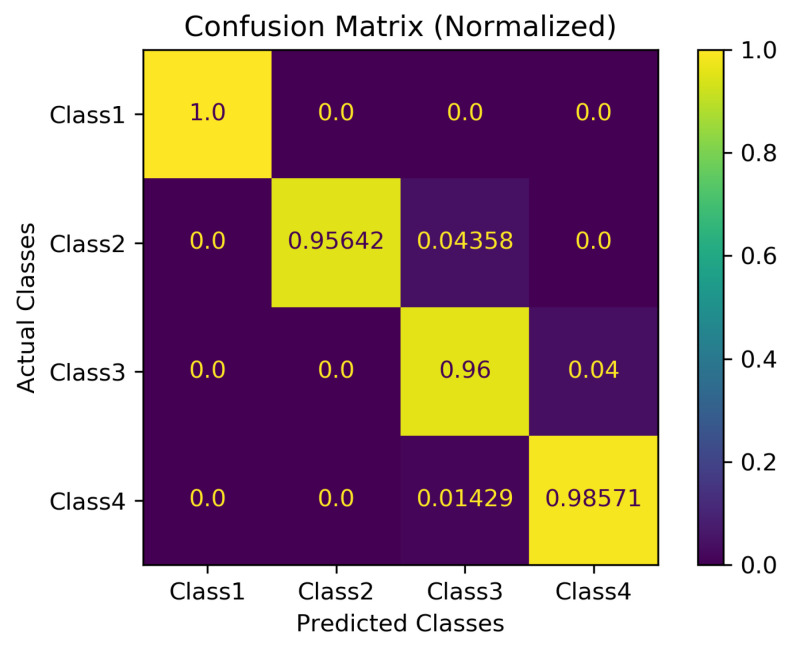
The normalized confusion matrix for 100 runs using 20 videos of the testing set. Classes 1, 2, 3, and 4 indicate unipedal stance, 8 ft up and go, 30 s chair stand, and 2 min step, respectively.

From the 100-run recognition test, the SO-ConvESN-ASHA achieved an average HAR accuracy of 97.1%, with a median of 100%. More than 50 runs of the tests achieved 100% accuracy. Compared to the baseline approach [59], which revealed an average accuracy of 91%, SO-ConvESN-ASHA accomplished about 6.7% improvement. Figure 20 shows the normalized confusion matrix of the overall recognition accuracy for the 100-run experiment. The Class 1 row shows that all 448 videos of “Unipedal Stance” were accurately classified with 100% accuracy. The Class 2 row achievs an accuracy of 95.64%, where 834 of 872 videos representing an “8 ft up and go” were correctly classified, and SO-ConvESN-ASHA wrongly classified 38 videos as “30 s chair stand”. The Class 3 row indicates an accuracy of 96% where 384 of 400 videos for “30 s chair stand” were correctly classified, and SO-ConvESN-ASHA wrongly classified 16 videos as a “2 min step”. The Class 4 row exhibits 98.57% accuracy, with 276 out of 280 were correctly classified as “2 min step”, and SO-ConvESN-ASHA wrongly classified four videos as “30 s chair stand”.

The results of the 100-run performance evaluation show that our proposed development framework, as shown in Figure 1, has successfully trained and optimized SO-ConvESN to recognize the fitness exercise actions with promising performance. In addition to the outperformed recognition accuracy, false recognition of SO-ConvESN-ASHA only shows up to one class. This achievement demonstrates the practicality of the proposed SO-ConvESN.

## 5. Conclusions

This paper presented a new method for 3D-skeleton-joints-based HAR by cascading a SORN-E into a simple CNN to yield SO-ConvESN. Current studies consider skeleton-joint human actions as multivariate time-series and attempt to identify and model the dynamical temporal features in 3D space. ESNs and their variants are a popular reservoir-computing method suitable for learning the temporal context. Following the body of work of ConvESN, it has incorporated modeling dynamics and multiscale temporal features in a unified framework. Despite the random initialization of the ESN’s input and reservoir, weights may reduce the computational cost, and on the other hand, this may raise instability and variance in generalization and hence diminish reproducibility. Moreover, hyperparameters of the ESN model are commonly tuned based on the rule of thumb or empirically fixed via trial-and-error by researchers’ past experiences. ESN also remains a black-box algorithm. Particularly, it lacks of explainability consideration to understand the input-dependent reservoir dynamics for HAR. To address these problems, we propose SORN-E, a novel self-organizing reservoir design approach that integrates ART architecture and topology construction based on ITM for learning node centroids and interconnectivity maps. To ensure stability and ESP in self-organizing reservoirs, we further exploit the RQA technique to explainability and characterize the dynamics of self-organizing reservoirs, hence tuning two critical ESN hyperparameters: input scaling and spectral radius. SORN-E is compatible with the deterministic initialization and self-organization of the ESN’s input and reservoir weights. The feature maps generated by SORN-E are applied to initialize the input weights and recurrent hidden weights in the ESN to yield optimized self-organizing reservoirs. We cascade the optimized self-organizing reservoirs with a simple CNN to learn the multiscale temporal features from ESRs for action recognition. This resultant novel implementation is named SO-ConvESN. With respect to the learning of the CNN stage, we also adopt three HPO algorithms: BO, ASHA, and PBT to optimize SO-ConvESN for HAR tasks. With the purpose of demonstrating the feasibility and applicability of the proposed approaches, we conduct experiments by using several publicly available 3D-skeleton-based action recognition datasets to examine the explainability of self-organizing reservoir dynamics, investigate the recognition accuracy of SO-ConvESN and the feasibility of implementing HPOs in SO-ConvESN for HAR task. The experimental results have demonstrated the applicability of SORN-E in deterministic initialization of ESN’s weights for reproducibility and generating optimized self-organizing reservoirs and the potentiality of the proposed SO-ConvESN in achieving competitive HAR performance compared with state-of-the-art approaches. Future works may consider unlocking the potential of SO-ConvESN for general time-series classification applications. Improvement of the robustness of the SO-ConvESN in noise handling capability may be another avenue for exploration and investigation.

## Figures and Tables

**Table 1:** Search Space Setup for MSRA3D Dataset.

Hyperparameter	Type	Values
Number of filters	Discrete	128, 256
Learning rate	Continuous	0.001, 0.003
Batch size	Discrete	1, 4

**Table 2:** Search Space Setup for Florence3D Dataset.

Hyperparameter	Type	Values
Number of filters	Discrete	256, 512
Learning rate	Continuous	0.001, 0.002
Batch size	Discrete	1, 4

**Table 3:** Search Space Setup for AHA3D Dataset.

Hyperparameter	Type	Values
Number of filters	Discrete	64, 128, 256
Learning rate	Continuous	0.001, 0.003
Batch size	Discrete	1, 4

**Table 4:** Performance Comparison of SO-ConvESN optimized by three different HPO Algorithms based on Validation Accuracy (%).

Algorithms	Baseline	BO	AHSA	PBT
MSRA3D Dataset	94.21	94.80	**95.09**	94.49
Florence3D Dataset	89.70	94.03	**96.07**	91.92

**Table 5:** Recognition accuracy on cross-subject test of the MSRA3D dataset.

Approaches	Average (%)
Covariance [63]	88.10
Skeletons Lie group [64]	92.40
DHMM+SL [65]	92.91
SO-ConvESN (Our approach)	94.21
SO-ConvESN-PBT (Our approach)	94.49
SO-ConvESN-BO (Our approach)	94.80
SO-ConvESN-ASHA (Our approach)	95.09
Gram matrices rep. [60]	96.90
ConvESN [10]	**97.88**

**Table 6:** Recognition accuracy on 10-fold cross-validation Florence3D-Action dataset.

Approaches	Average (%)
Multi-Part Bag-of-Poses [58]	82.00
SO-ConvESN (Our approach)	89.70
Skeletons Lie group [64]	90.88
ConvESN [10]	91.72
SO-ConvESN-PBT (Our approach)	91.92
SO-ConvESN-BO (Our approach)	94.03
SO-ConvESN-ASHA (Our approach)	96.07
Complete GR-GCN [66]	98.40
Deep STGC${}_{K}$ [67]	**99.10**

## Data Availability

Data available in a publicly accessible repository that does not issue DOIs. Publicly available datasets were analyzed in this study. This data can be found here: [https://sites.google.com/view/wanqingli/data-sets/msr-action3d] https://www.micc.unifi.it/resources/datasets/florence-3d-actions-dataset/; https://vislab.isr.tecnico.ulisboa.pt/datasets/ (accessed on 24 September 2019).

## Abbreviations

The following abbreviations are used in this manuscript:

AHA3D	Augmented Human Assistance 3D
ART	Adaptive Resonant Theory
ASHA	Asynchronous Successive Halving Algorithm
BO	Bayesian Optimization
ConvESN	Convolutional Echo State Network
CT	Central Trunk
DET	Determinism level
EI	Expected Improvement
ESNs	Echo State Networks
ESP	Echo State Property
Florence3D	Florence 3D Actions
GNG	Growing Neural Gas
HPO	Hyperparameter Optimization
HAR	Human Action Recognition
ITM	Instantaneous Topological Mapping
LA	Left Arm
LAM	Laminarity
LI-ESNs	Leaky Integrator-Echo State Networks
LL	Left Leg
$L_{MAX}$	Maximum diagonal line length
LSTM	Long Short-Term Memory
MSRA3D	Microsoft Research Action 3D
NG	Neural Gas
PBT	Population-based Training
RA	Right Arm
RC	Reservoir Computing
ReLU	Rectified Linear Unit
RL	Right Leg
RNNs	Recurrent Neural Networks
RPs	Recurrent Plots
RQA	Recurrence Quantification Analysis
RR	Recurrence Rate
SHA	Successive Halving Algorithm
SO-ConvESN	Self-Organizing Convolutional Echo State Network
SOM	Self-Organizing Map
SORN	Self-Organizing Reservoir Network
SORN-E	Self-Organizing Reservoir Network with Explainability
